# Large-diameter titanium dioxide nanotube arrays as a scattering layer for high-efficiency dye-sensitized solar cell

**DOI:** 10.1186/1556-276X-9-362

**Published:** 2014-07-20

**Authors:** Xiaolin Liu, Min Guo, Jianjun Cao, Jia Lin, Yuen Hong Tsang, Xianfeng Chen, Haitao Huang

**Affiliations:** 1 The State Key Laboratory of Advanced Optical Communication Systems and Networks, Department of Physics, Shanghai Jiao Tong University, 800 Dongchuan Road, Shanghai 200240, China; 2 Department of Applied Physics, The Hong Kong Polytechnic University, Hung Hom, Kowloon, Hong Kong

**Keywords:** Large-diameter titanium dioxide nanotubes, Scattering layer, Dye-sensitized solar cells, Electrochemical reactions

## Abstract

Large-sized titanium dioxide (TiO_2_) nanotube arrays with an outer diameter of approximately 500 nm have been successfully synthesized by potentiostatic anodization at 180 V in a used electrolyte with the addition of 1.5 M lactic acid. It is found that the synthesized large-diameter TiO_2_ nanotube array shows a superior light scattering ability, which can be used as a light scattering layer to significantly enhance the efficiency of TiO_2_ nanoparticle-based dye-sensitized solar cells from 5.18% to 6.15%. The remarkable light scattering ability makes the large-diameter TiO_2_ nanotube array a promising candidate for light management in dye-sensitized solar cells (DSSCs).

## Background

Dye-sensitized solar cells (DSSCs) have received considerable interest since 1991 [1] with the growing concern on sustainable and renewable energies. The highest power conversion efficiency (PCE) of DSSCs based on TiO_2_ nanoparticle mesoporous films has been reported [2], and to further improve the PCE, plenty of research has been carried out, such as the development of new dyes with broadband absorption [3,4], the increase of the sensitized surface area of the TiO_2_ film [5,6], and the use of a scattering layer for enhanced light harvesting [7-13]. Among them, the introduction of a scattering layer with different structures has been widely studied and proven to be effective in light harvesting enhancement. TiO_2_ nanorods with a length of 180 to 250 nm have been used as scattering centers in DSSCs by Yoon et al. [9]. Liu et al. had dispersed TiO_2_ nanospheres into nanocrystallites for increased light harvesting in DSSCs [10].

However, scattering centers of large-scale micrometer particles embedded in the absorbing layer of DSSCs would reduce the dye loading amounts. Hence, a bi-layer structure with the scattering layer beneath the absorbing layer to increase the optical path length is more favorable. Hierarchical TiO_2_ hollow spheres with an outer diameter of 300 to 700 nm [11] and size-tunable mesoporous spherical TiO_2_[12] have been tried as the scattering layer in bi-layer-structured DSSCs.

While the scattering of nanofibers and nanotubes was found to satisfy the Mie theory, which was originally proposed to describe the scattering of particles of a size similar to the wavelength [13-15], there are only few relevant reports on applying TiO_2_ nanotubes with a subwavelength-sized diameter as the scattering layer. Herein, we succeeded in a straightforward approach to the fabrication of large-diameter (comparable to wavelength) TiO_2_ nanotubes and characterized the light scattering effect by transmittance spectra measurement and finite-element full wave simulation. The anodization was processed at 180 V in a used electrolyte with the addition of 1.5 M lactic acid. The transmittance spectra showed that the large-diameter nanotubes have a superior light scattering property. When the large-diameter TiO_2_ nanotube membrane was successfully peeled off and used as the scattering layer in DSSCs, the PCE was found to increase from 5.18% to 6.15% under 1 Sun (or 5.23% to 6.36% under 0.5 Sun) and increased by 19% (or 22%) due to the strong light scattering of large-diameter TiO_2_ nanotubes.

## Methods

The large-diameter TiO_2_ nanotubes were fabricated through potentiostatic anodization in a conventional two-electrode electrochemical cell. Titanium sheets (0.125 mm in thickness, Strem Chemicals, Newburyport, MA, USA) were used as the working electrode while Pt foil was the counter electrode, with the distance between electrodes being 2 cm. The anodization process was divided into three steps: (1) The Ti foil was electrochemically pretreated for 0.5 h at 60 V in an ethylene glycol electrolyte containing 0.5 wt% NH_4_F and 3 vol% H_2_O (anodization electrolyte #1). After anodization, the anodized layer was peeled off by intense ultrasonication to expose the substrate. (2) The surface-exposed Ti was processed in another ethylene glycol electrolyte with 0.5 wt% NH_4_F and 10 vol% H_2_O, added with 1.5 M lactic acid (LA) (anodization electrolyte #2). Electrolyte #2 was aged by anodization reaction at 60 V for about 10 h before usage. To fabricate large-diameter nanotubes, the anodization voltage was fixed at 120 V for 10 min and then gradually increased to 180 V for 10 min at a rate of 0.1 V/s. (3) The as-grown large-diameter nanotubes were annealed at 450°C for 2 h and then detached from the Ti substrate by a third anodization at 60 V in electrolyte #1 to obtain the freestanding membranes [16]. For comparison, freestanding TiO_2_ nanotube membranes of the same thickness but with smaller diameters were also fabricated by anodization at 60 V for 10 min in electrolyte #1.

The resulting nanotube membrane was used as a scattering layer by adhering to the fluorine-doped tin oxide (FTO) substrate with TiO_2_ NP paste via a doctor blade method, followed by sintering at 450°C for 2 h. The sintered photoanodes were immersed in a dye-containing solvent (N719 dye, Dyesol, Queanbeyan, New South Wales, Australia) for 3 days. A 25-μm-thick hot-melt spacer was used to separate the sensitized photoanode and the counter electrode which was prepared by thermal decomposition of H_2_PtCl_6_ isopropanol solution on FTO glass at 380°C for 30 min. The interspace was filled with a liquid electrolyte of DMPII/LiI/I_2_/TBP/GuSCN in 3-methoxypropionitrile.

The structure and morphology of the TiO_2_ nanotubes were analyzed using field-emission scanning electron microscopy (FESEM; JEOL JSM-6335 F, JEOL Ltd., Tokyo, Japan). The current density-voltage (*J*-*V*) characteristics were measured by a sourcemeter (Model 2420, Keithley Instruments, Inc., Cleveland, OH, USA) under AM 1.5G illumination (100 mW cm^−2^) which was provided by a 300-W solar simulator (Model 91160, Newport Corporation-Oriel Instruments, Irvine, CA, USA). Transmittance spectra were acquired using a UV–Vis spectrophotometer (Model UV-2550, Shimadzu Corporation, Tokyo, Japan). The amount of dye was measured by desorbing the attached dye molecules in 0.1 M NaOH aqueous solution, with the concentration determined by a UV–Vis spectrophotometer. The normalized incident photon-to-current conversion efficiency (IPCE) values were measured with an IPCE system equipped with a xenon lamp (Oriel 66902, 300 W), a monochromator (Newport 66902), and a dual-channel power meter (Newport 2931_C) equipped with a Si detector (Oriel 76175_71580).

## Results and discussion

**Figure 1:**
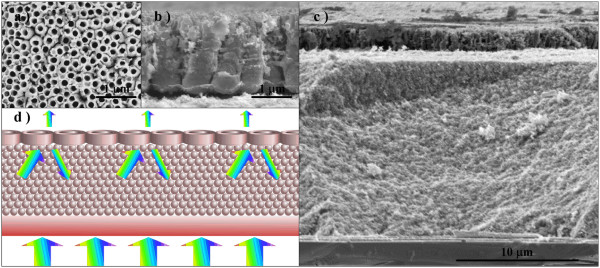
**SEM images and schematic of the photoanode. (a)** Top and **(b)** cross-sectional SEM images of LTNAs. **(c)** Cross-sectional SEM image of the LTNA as a scattering layer on top of TiO_2_ nanoparticles. **(d)** Schematic of the photoanode structure with scattered incident light.

Shown in Figure 1a,b are top and cross-sectional SEM images of the large-diameter TiO_2_ nanotube arrays (LTNAs). As reported before, the nanotube diameter is determined by the water content in the electrolyte and the anodization voltage, with a larger diameter obtained under more water content and higher voltage [17,18]. Meanwhile, the addition of LA and the use of an aged electrolyte can prevent the anodic breakdown and the oxide burning under too large a current density at high anodization voltages [19,20]. In the second step of the anodization process, prior to the anodization at 180 V, a pretreatment at 120 V for 10 min was adopted to maintain a flat anodic TiO_2_ film surface. With this pretreatment, the surface diameter was smaller than that at the bottom of the nanotubes. As can be seen from Figure 1a,b, the diameters of LTNA are approximately 500 nm at the bottom and approximately 300 nm at the surface. The nanotubes have a typical length of approximately 1.8 μm, with roughened tube walls. For comparison, we also fabricated small-diameter TiO_2_ nanotube arrays (STNAs) with a diameter of approximately 120 nm, which were anodized at 60 V.

**Figure 2:**
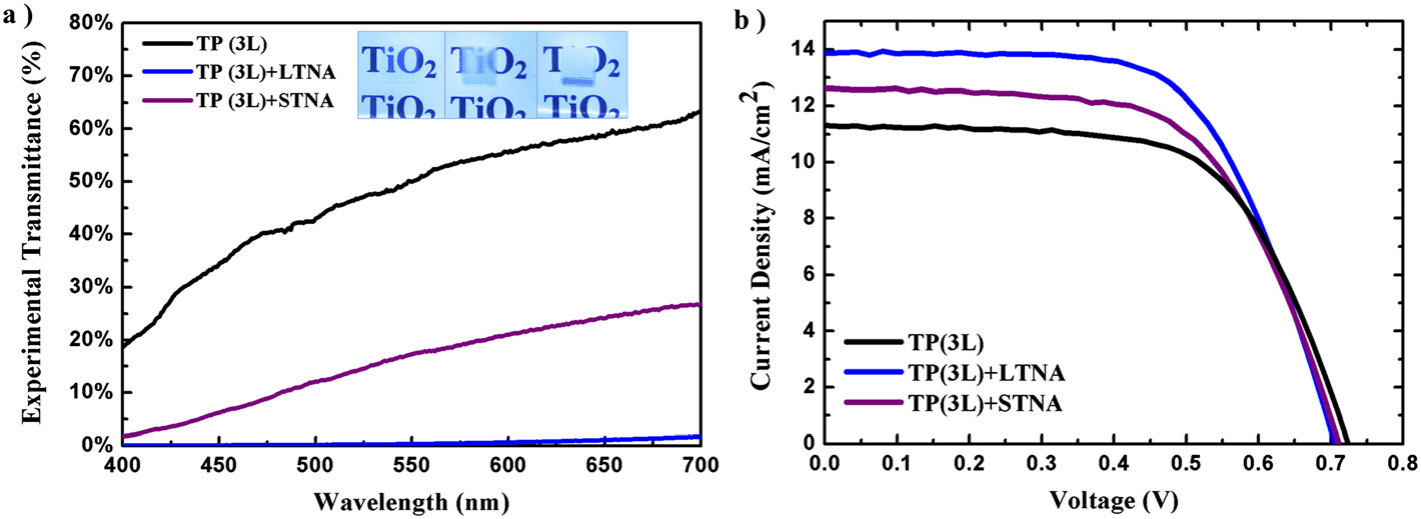
**Optical and photovoltaic properties. (a)** Transmittance of the three types of photoanodes adhered to the FTO glass substrates before the sensitization with N719. The insets from left to right show the photos of the photoanodes, TP (3 L), TP (3 L) + STNA, and TP (3 L) + LTNA, respectively. Here, 3 L stands for the optimized thickness of the TiO_2_ particle layer in a TP-based DSSC. **(b)** Photocurrent-voltage curves (1 Sun) of the TP (3 L)-based DSSCs coupled with different scattering layers, i.e., LTNA and STNA, with a thickness of 1.8 μm.

The light scattering effect was characterized by measuring the transmittance spectra of three types of photoanodes adhered to FTO glass substrates (Figure 2a), namely, TiO_2_ particles (TP), TP + STNA, and TP + LTNA. It can be seen clearly that LTNA has a superior light scattering property than STNA, as the TP + LTNA sample is opaque and the TP + STNA sample is semitransparent. The TP sample is the most transparent, with the highest transmittance in the visible range. Finite-element full wave simulation (Additional file 1: Figure S1) was used to numerically calculate the transmittance spectra of the two different types of TNAs [21,22], which revealed that light propagates through STNA without remarkable scattering, while pronounced scattering occurs in LTNA. The high anodization voltage also enables the formation of some randomly orientated nanotubes and defects [23], which further enhance the light scattering in LTNA. Hence, experimentally, the TP + LTNA sample shows extremely low transmittance in the whole visible range (Figure 2a).

To study the effect of the scattering layer on the PCE of DSSC, the thickness of the TiO_2_ particle layer was first optimized by measuring the PCE of five TP-based DSSCs in different thicknesses (Additional file 1: Figure S2). The PCE was found to increase from 3.52% for TP (1L) to 5.18% for TP (3L) due to increased thickness (from 5 to 14 μm). It then starts to decrease when the TP layer thickness was further increased. The sample with the optimized thickness, TP (3L), was chosen to be attached to the STNA and LTNA scattering layers, with a thickness of around 1.8 μm as shown in Figure 1c,d. At least four cells were tested for each type of the solar cells, and their representative *I*-*V* curves are shown in Figure 2b and Table 1 with the photovoltaic properties. It is found that both *η* and *J*_SC_ were enhanced due to the attachment of a scattering layer. The *J*_SC_ is increased from 11.3 mA cm^−2^ for the TP (3L) cell to 13.9 mA cm^−2^ for the TP (3L) + LTNA cell. Due to the higher light scattering power of the LTNA than that of the STNA, the percentage increase in *η* is approximately 19% (from 5.18% to 6.15%) for the TP (3L) + LTNA cell, higher than the approximately 6.5% increase for the TP (3L) + STNA cell. It is also noted that due to the attachment of the scattering layer, the dye loading amount was increased. However, the increased dye loading contributes less to the increase of *η* than the enhanced light scattering does due to the fact that the TP layer thickness has already been optimized. Further increase in the thickness of the photoanode will result in a decrease in *η*, though the dye loading is increased. Indeed, although the TP (3L) + STNA cell has a higher dye loading than the TP (3L) + LTNA one, its *η* is much lower (Table 1). This further demonstrates the importance of light scattering.

**Table 1:** Photovoltaic properties of the DSSCs with and without the scattering layers

**Samples**	**TiO**_**2 **_**thickness (μm)**	***J***_**SC **_**(mA cm**^**−2**^**)**	***V***_**OC **_**(V)**	**FF**	**Relative dye loading**	***η *** **(%) 1 Sun**	***η *** **(%) 0.5 Sun**
**TP (3 L)**	14	11.32	0.724	0.632	0.342	5.18	5.23
**TP (3 L) + LTNA**	14 + 1.8	13.87	0.705	0.629	0.446	6.15	6.36
**TP (3 L) + STNA**	14 + 1.8	12.63	0.711	0.614	0.457	5.52	5.64

**Figure 3:**
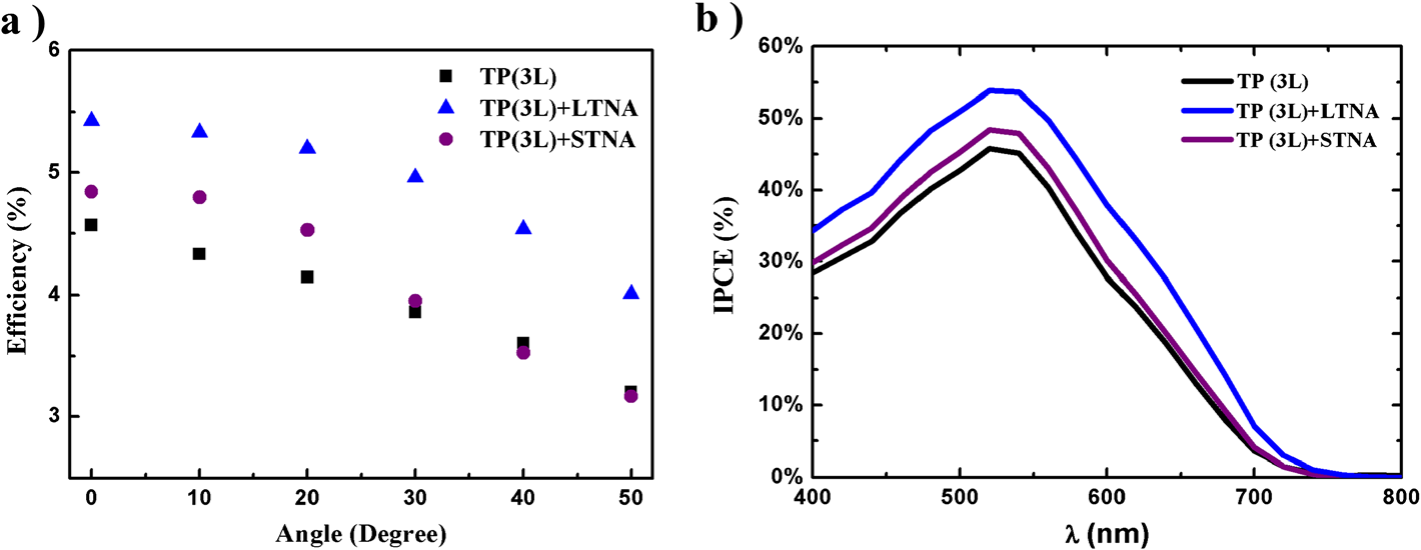
**DSSC angle performance and IPCE. (a)** Variation of efficiency with the angle of incidence of incoming light with respect to the three types of cells. **(b)** IPCE of the TP (3 L)-based DSSCs coupled with different scattering layers, i.e., LTNA and STNA.

The *I*-*V* curves of the three types of DSSCs under lower irradiation (0.5 Sun) were also measured (Additional file 1: Figure S3). Owing to the excellent scattering property of the LTNA layer, an efficiency of 6.36% was achieved in the TP (3L) + LTNA cell, in comparison with the efficiencies of 5.23% and 5.64% achieved in the TP (3L) and TP (3L) + STNA cells, respectively. The angular response of the three types of DSSCs was also investigated and compared (Figure 3a). Due to the high scattering power of the LTNA layer for the different photon propagation directions, the enhanced light absorption effect is less sensitive to the tilting of the cells.

The incident photon-to-current conversion efficiency (IPCE) spectra are depicted in Figure 3b to provide detailed information on light harvesting. It is observed that the main light harvesting enhancement caused by the scattering layer occurs not only in the dye absorption range but also in the long wavelength side [24,25], which is exactly the wavelength range for the small dye absorption. Consequently, the TP (3L) + LTNA cell is able to more efficiently recapture the unabsorbed light which resulted from the efficient light scattering capability of the LTNA layer. A further insight into the electrochemical behavior was provided by the EIS measurement in the dark at different applied bias voltages. The electron recombination time (*τ*_n_) was calculated from the Bode phase plots by *τ*_n_ = 1/(2*πf*_peak_), where *f*_peak_ is the characteristic peak frequency in the mid-frequency (1 to 100 Hz) region [5,26]. As shown in Additional file 1: Figure S4, the use of the light scattering layer does not significantly influence the *τ*_n_ and hence does not affect the electron transport.

## Conclusions

Large-diameter TiO_2_ nanotube arrays were successfully synthesized. The outstanding scattering power of the LTNA layer was demonstrated by the transmittance spectra and the optical simulation. The LTNA layer is superior to the STNA one in terms of light scattering. The use of the LTNA as the scattering layer in DSSCs enhances the PCE (from 5.18% to 6.15%) and the short-circuit current density much more than the STNA does. It is believed that the large-diameter nanotubes can be applied to other types of solar cells and higher conversion efficiency can be achieved by further optimization.

## Competing interests

The authors declare that they have no competing interests.

## Authors’ contributions

XC and HH proposed the idea and presided over the study. XL, MG, JC, and YT conceived and designed the experiment. XL and JL wrote the paper. All authors read and approved the final manuscript.

## Supplementary Material

Additional file 1 **Supporting information.** Figure S1 The normalized and simulated transmittance spectra of the three types of photoanodes adhered to the FTO glass substrates before the sensitization with N719. Figure S2 (a) Photocurrent-voltage curves and (b) photovoltaic properties of the TP based DSSCs with different thickness. Figure S3 (a) Photocurrent-voltage curves under 0.5 Sun and (b) photovoltaic properties of the TP(3 L) based DSSCs coupled with different scattering layers, i.e., LTNA and STNA with the same thickness of 1.8 μm. Figure S4 Electron lifetime of three types of DSSCs in the dark at different applied bias voltages. Click here for file
