# Supplementary material for: Large-diameter titanium dioxide nanotube arrays as a scattering layer for high-efficiency dye-sensitized solar cell
Source: Nanoscale Res Lett. 2014 Jul 20;9(1):362. doi: 10.1186/1556-276X-9-362 (PMC4108594; doi:10.1186/1556-276X-9-362)
Supplement: Additional file 1 — Supporting information. Figure S1 The normalized and simulated transmittance spectra of the three types of photoanodes adhered to the FTO glass substrates before the sensitization with N719. Figure S2 (a) Photocurrent-voltage curves and (b) photovoltaic properties of the TP based DSSCs with different thickness. Figure S3 (a) Photocurrent-voltage curves under 0.5 Sun and (b) photovoltaic properties of the TP(3 L) based DSSCs coupled with different scattering layers, i.e., LTNA and STNA with the same thickness of 1.8 μm. Figure S4 Electron lifetime of three types of DSSCs in the dark at different applied bias voltages. [file 1556-276X-9-362-S1.doc]

**Additional file 1**

**Supporting Information**

**Figure legends**


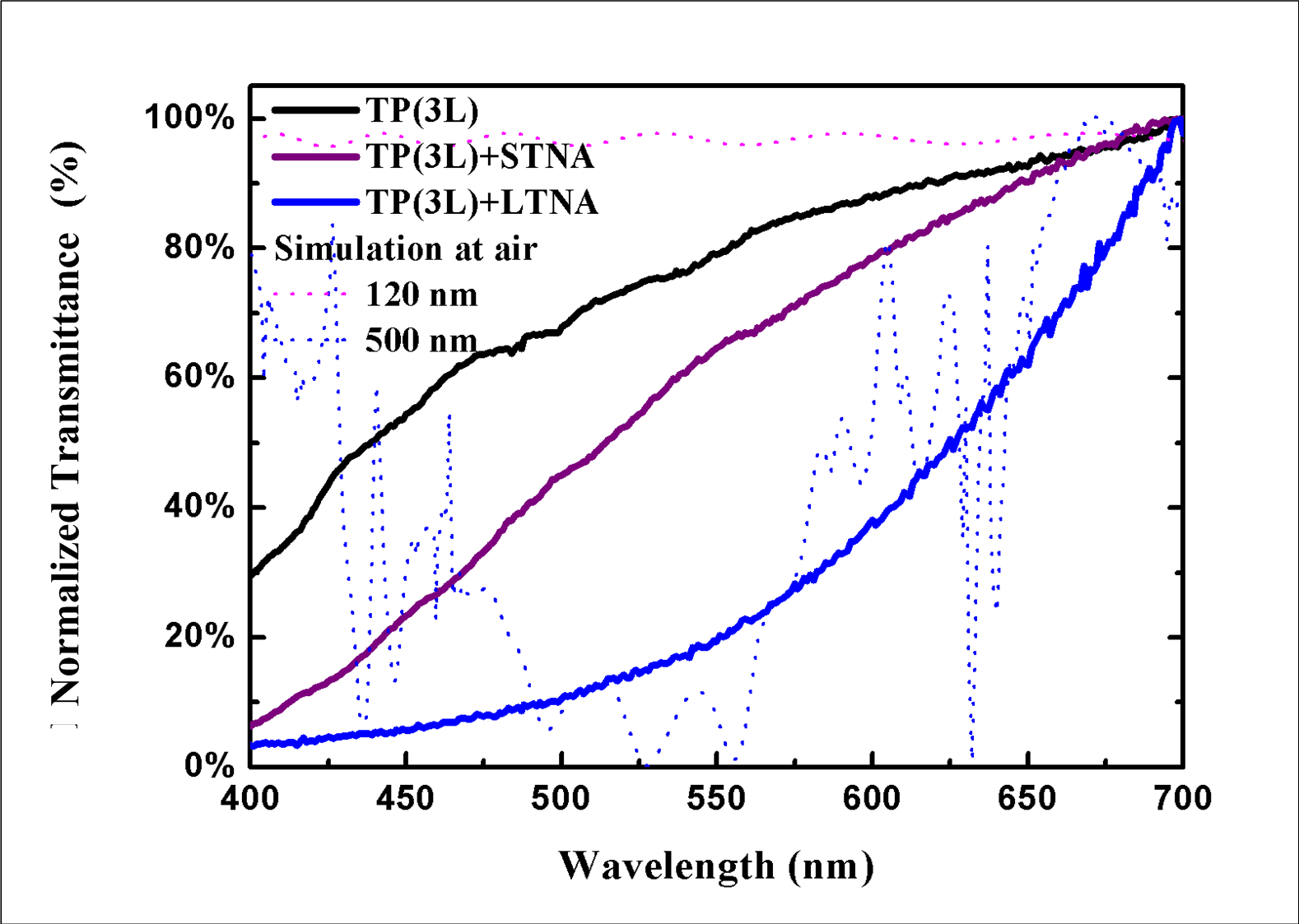


**Figure S1** The normalized and simulated transmittance spectra of the three types of photoanodes adhered to the FTO glass substrates before the sensitization with N719.

The numerical calculations of the transmittance spectra of two different types of TNAs were employed by finite-element full wave simulation. The TiO_2_ nanotubes were arranged in a hexagonal lattice with the outer diameter, the wall thickness and the tube length being 500 nm, 50 nm, 1.8 μm, respectively, for the LTNA and 120 nm, 10 nm, 1.8 μm, respectively, for the STNA, according to the SEM images.


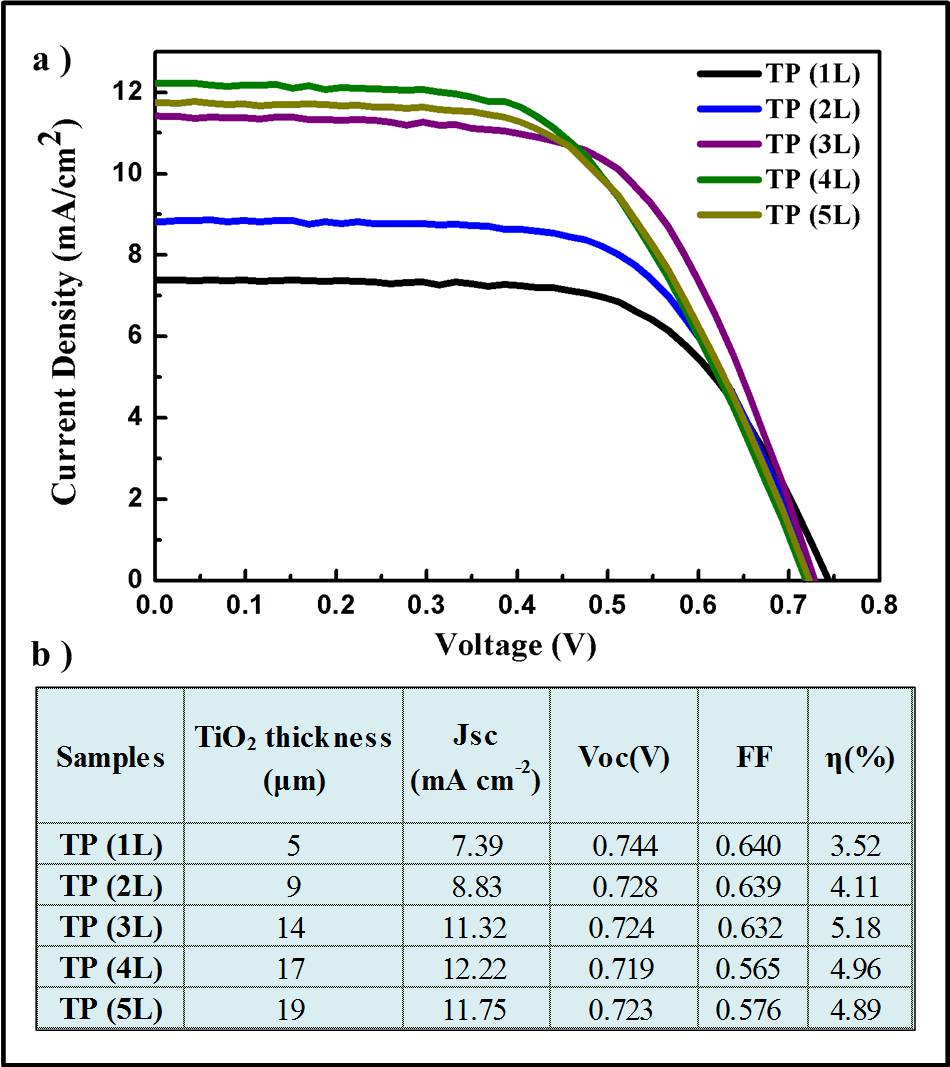


**Figure S2** (a) Photocurrent-voltage curves and (b) photovoltaic properties of the TP based DSSCs with different thickness.


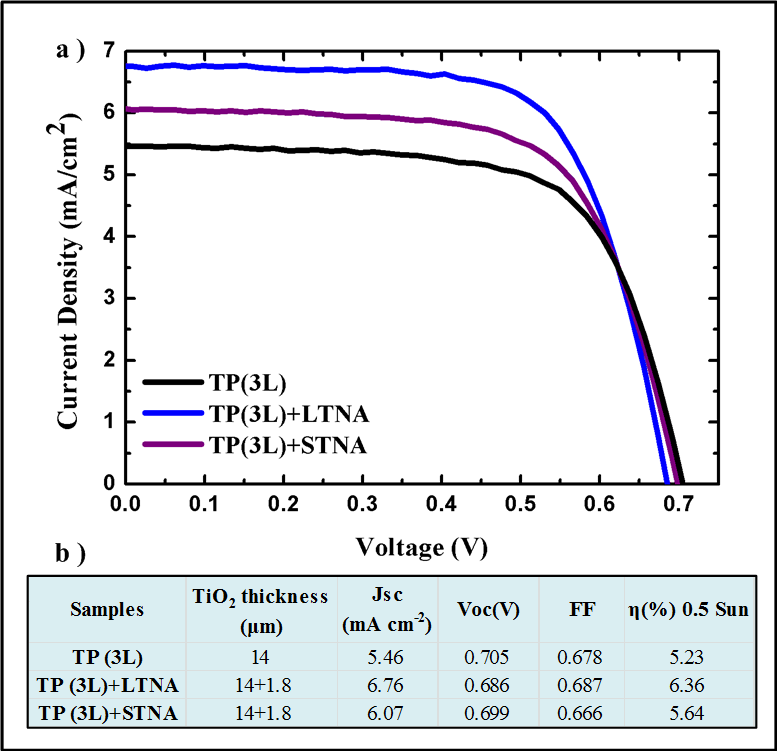


**Figure S3** (a) Photocurrent-voltage curves under 0.5 Sun and (b) photovoltaic properties of the TP(3L) based DSSCs coupled with different scattering layers, *i.e.*, LTNA and STNA with the same thickness of 1.8 μm


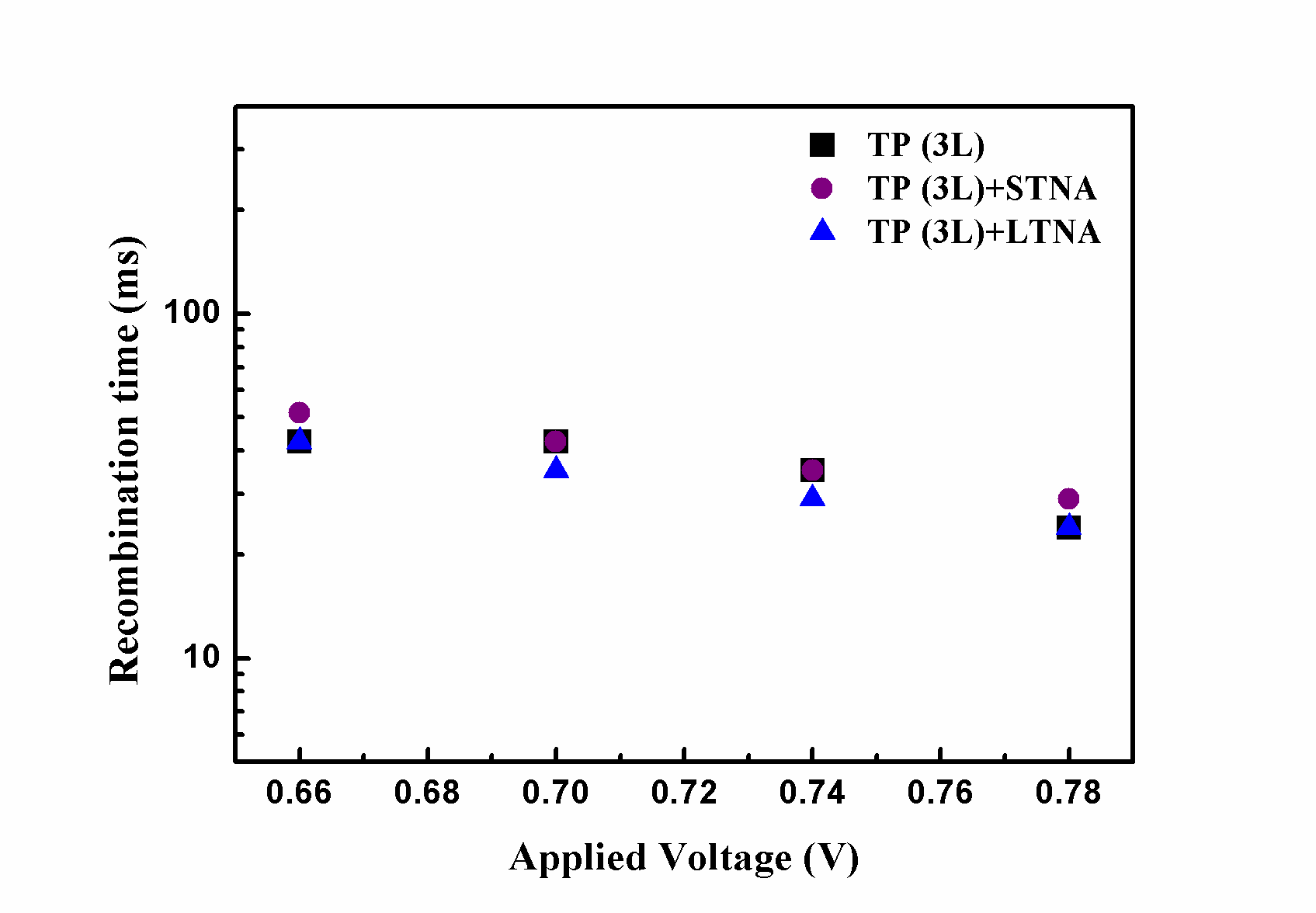


**Figure S4** Electron lifetime of three types of DSSCs in the dark at different applied bias voltages
